# The fog of war: How network buffering protects plants’ defense secrets from pathogens

**DOI:** 10.1371/journal.pgen.1006713

**Published:** 2017-05-04

**Authors:** Brett M. Tyler

**Affiliations:** Center for Genome Research and Biocomputing and Department of Botany and Plant Pathology, Oregon State University, Corvallis, Oregon, United States of America; University of California Davis, UNITED STATES

Plants lack the mechanisms needed for adaptive immunity and thus rely entirely on innate immunity to protect themselves from pathogens and pests [1]. Their innate immune systems must protect against innumerable, highly adaptable viruses bacteria, fungi, oomycetes, nematodes, and insects. Two important components of the plant immune system are induced by the presence of pathogen molecules [1]. One of these is triggered by molecules common to multiple microbes (pathogen-associated molecular patterns [PAMPs]), such as bacterial flagellin, fungal, or arthropod chitin fragments. PAMP-triggered immunity (PTI) is mediated by a diversity of cell surface receptors, some of which carry intracellular kinase domains. The second inducible component of defense is triggered as a result of plants’ recognition of specific virulence proteins (effectors) produced by pathogens—known as effector-triggered immunity (ETI).

Complex and overlapping networks of signal transduction events mediate and integrate the induction of the defense responses that comprise PTI and ETI [2]. Signaling networks that mediate plants’ responses to the abiotic environment must also be integrated [3]. These signaling networks involve phosphorylation cascades as well as the release of chemical signals, such as reactive oxygen species, lipid and inositol derivatives, and plant hormones, such as salicylic acid (SA), jasmonic acid (JA), ethylene (ET), and abscisic acid [4]. The defense responses themselves include thousands of gene expression changes involving genes encoding antimicrobial proteins and peptides, secondary metabolite biosynthetic genes, and many genes of currently unknown function [4].

Molecular dissections of successful plant pathogens have revealed that a common strategy for overcoming plant defenses is the production of chemicals and/or effector proteins that can enter inside host cells and interfere with the signaling events responsible for PTI and ETI [5]. A major challenge for plants therefore is to protect these signaling events from pathogen interference.

**Fig 1:**
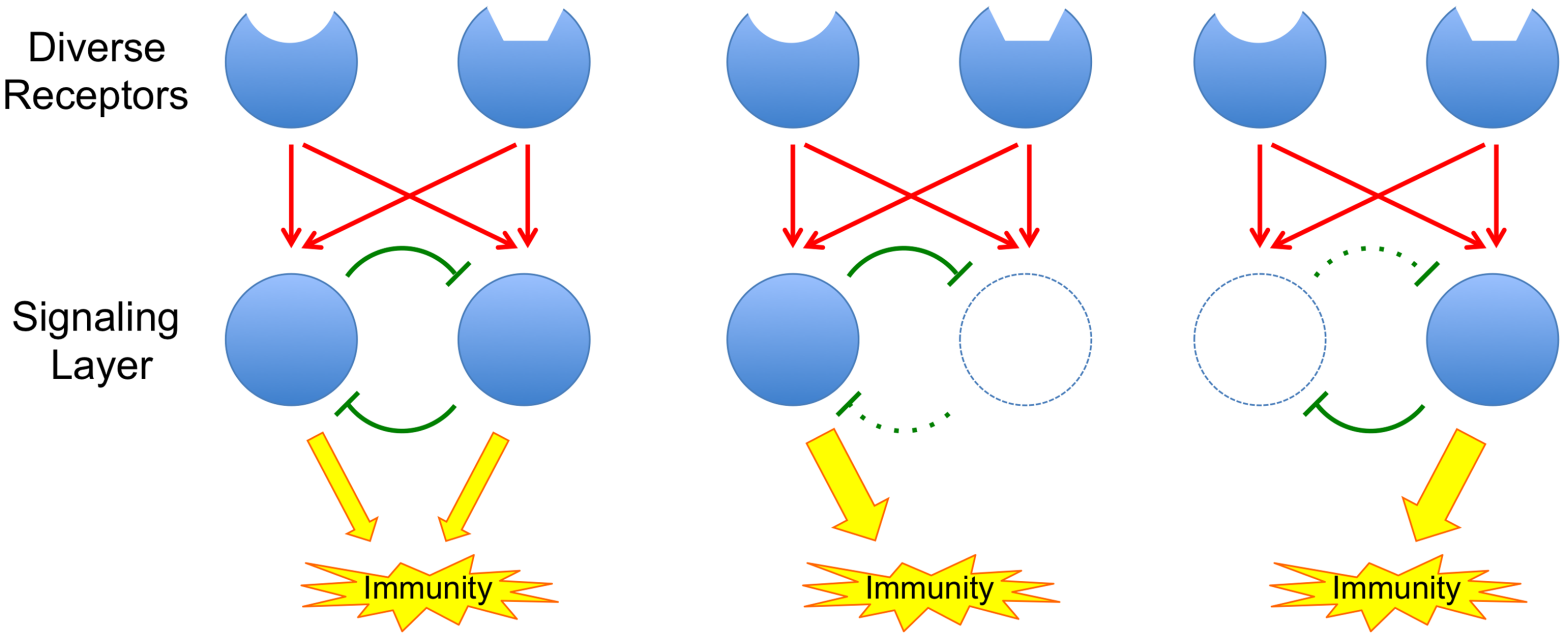
The principle of a buffered signaling network. Red arrows represent activation and green blunt arrows represent inhibition. Inhibitory loops within the network provide buffering; when network components are attacked, release of associated inhibitory loops allows other components of the network to compensate for the loss.

In this issue of *PLOS Genetics*, Hillmer et al. [6] describe a highly detailed, systems-level dissection of the signaling network that mediates PTI responses in the model plant *Arabidopsis*. The study, which integrates mutational analysis, transcript and hormone profiling, and statistical modeling, reveals that the PTI signaling network is highly buffered against interference. In other words, overall transmission of signaling through the network persists even when major components are interrupted, for example by mutations or by the actions of pathogen effectors (Fig 1). Buffering of the immune signaling network can also influence pathogen evolution as it obscures the contributions of individual pathogen proteins to virulence.

This work builds on two previous papers from the Katagiri lab. In 2009, Tsuda et al. [7] showed that mutations in the key *Arabidopsis* genes, *DDE*2, *EIN*2, *SID*2, and *PAD*4, defined four major sectors of the immune signaling network. Mutations in any one gene had little effect on immunity, but together, mutations in all four genes abolished around 80% of the plant’s immunity to two pathogens. Mutations in *DDE*2, *EIN*2, and *SID*2 defined sectors associated with the plant hormones JA, ET, and SA, respectively, while *PAD*4 mutations defined a new sector not associated with any known hormones. By quantitating the level of immunity remaining in all possible single, double, triple, and quadruple mutants, following stimulation with a selection of PAMPs and effectors, Tsuda et al. [7] could use a statistical modeling approach (signal allocation via a mixed general linear model) to assess how signaling information flowed through the network. Their analysis revealed strong interactions among the components of the network, including additive, synergistic, and compensatory interactions. The compensatory interactions are of particular importance as they are responsible for the buffering or resilience of the network against external interference (Fig 1).

In 2014, Kim et al. [8] examined gene expression changes underlying signal flow through the network. They used four genes that were controlled only by one sector (JA, ET, SA, and PAD4) as readouts of individual sector activity during stimulation by two PAMPs (flg22 and chitosan) in all 16 mutant genotypes. The results suggested that interactions among the four sectors create a four-point switch, which is resilient to external interference, but nevertheless can be flipped into different states as needed to adapt to challenges from pathogens with different lifestyles, such as biotrophs or necrotrophs.

In the group’s latest paper, Hillmer et al. [6] expand the analysis to examine how the *Arabidopsis* broader transcriptome and hormone repertoires respond to the activities of the four sectors during PTI, using the flagellin fragment flg22 as a model PAMP. The transcriptome was assessed by tag-sequencing, while the levels of 44 plant hormones, including SA and JA were measured via liquid chromatography-mass spectrometry. The transcriptome and hormones were measured at 7 time points in 18 genotypes (the 16 combinations of *dde*2, *ein*2, *sid*2, and *pad*4 mutations plus wild type and a mutation in the flg22 receptor gene, *FLS*2). Two hormones (SA and JA) and 5,259 genes exhibited responses to flg22 that were statistically significant and also significantly perturbed in at least one genotype. Signal allocation analysis (as in Tsuda et al. [7]) was then carried out to determine which of the four sectors was responsible for regulating each gene/hormone and included all possible interactions between sectors. Interaction was defined as any combined effect of two or more sectors that was not simply additive (on a log scale). An example of an interaction is synergy, which is when the response to two sectors combined is stronger than the sum of the responses to the two sectors individually, as assessed by comparing relevant mutants that retain one or both of the sectors in question. Another important interaction is buffering or compensation, which is observed when the response to two sectors combined is weaker than the sum of the responses to the two sectors individually.

The results revealed that interactions played a major role in regulating the genes and hormones—99.5% of the genes and hormones had a least one significant interaction component of their regulation. In 64% of genes, regulation by interactions outweighed regulation by individual sectors. Furthermore, in 61% of cases, the combined regulation by interactions was in the opposite direction than the combined effectors of the individual sectors, which indicated that buffering was widespread in the regulatory network. Extensive buffering was also evident when the effects of the single sector mutations on transcript levels were compared to the effects of the four-sector knockout. Fifty-seven percent of genes were fully buffered (i.e., the single mutations had no effect) and 30% were even more deeply buffered, because they remained unaffected by any double mutation.

The highly buffered nature of the immune response network provides it with a strong ability to resist interference by pathogen effectors or toxins that may target individual components of the network (Fig 1). Only pathogens that have evolved a sufficiently comprehensive repertoire of effectors and toxins that can blanket the entire signaling network will succeed in causing disease.

Additionally, the buffering of the network makes it resistant to accurate dissection by conventional genetic approaches that rely on the isolation of single gene mutations. As pointed out by Hillmer et al. [6], a more effective way to analyze a system such as this is a network reconstruction approach in which multiple mutations are used to reduce the system to its ground state, and then sectors are restored one by one or in combinations by removal of mutations.

A buffered host immune system has important implications for pathogen evolution. If the function provided by the host target of an effector is buffered, then selection for retaining that effector gene in the pathogen genome will be greatly weakened. Similarly, a mutation that enables an effector to acquire a new target will not be selected for if the function of that target is buffered. Thus, in military terms, host plants can confuse pathogens’ weapons development programs by effectively hiding the damage caused by existing and new weapons.

The same consideration also affects researchers’ ability to identify key pathogen virulence genes—if the host target of a virulence gene is buffered, then the pathogen gene will appear to be dispensable; this may explain numerous reports of pathogen genes that have obvious virulence-related functions but appear dispensable when individually deleted or silenced [9,10]. Till now, dispensable pathogen genes have been interpreted to indicate redundancy in the pathogen, but buffering of the targeted host function must now be considered as an alternative explanation.

Although admirably comprehensive in its design and scope, the study by Hillmer et al. [6] still has peeled back just one layer of the *Arabidopsis* defense signaling network, namely the response to flg22. Considering the vast complexity of pathogen molecules exposed to the plant during a natural infection, as well as molecules originating from other members of the plant’s microbiome, much remains to be learned about the way that the immune network integrates a plethora of inputs.
